# Current Standards for and Clinical Impact of Emergency Radiology in Major Trauma

**DOI:** 10.3390/ijerph19010539

**Published:** 2022-01-04

**Authors:** Francesca Iacobellis, Ahmad Abu-Omar, Paola Crivelli, Michele Galluzzo, Roberta Danzi, Margherita Trinci, Giuseppina Dell’Aversano Orabona, Maurizio Conti, Luigia Romano, Mariano Scaglione

**Affiliations:** 1 Department of General and Emergency Radiology, “A. Cardarelli” Hospital, A. Cardarelli St. 9, 80131 Naples, Italy; giuseppinadellaversanoorabona@gmail.com (G.D.O.); luigia.romano1@gmail.com (L.R.); 2 Department of Radiology, The James Cook University Hospital, Middlesbrough TS4 3BW, UK; aomar@doctors.org.uk (A.A.-O.); mscaglione@tiscali.it (M.S.); 3 Department of Clinical and Experimental Medicine, University of Sassari, Via Roma 151, 07100 Sassari, Italy; paocri2000@gmail.com (P.C.); mconti@uniss.it (M.C.); 4 Department of Radiology, Azienda Ospedaliera San Camillo Forlanini, C.Ne Gianicolense, 87, 00152 Rome, Italy; galluzzom@tiscali.it (M.G.); margherita.trinci@libero.it (M.T.); 5 Department of Radiology, Pineta Grande Hospital, Via Domitiana Km 30, 81030 Castel Volturno, Italy; danziroberta@gmail.com; 6 School of Health and Life Sciences, Teesside University, Middlesbrough TS1 3BX, UK; 7 Italian Society of Medical and Interventional Radiology (SIRM), SIRM Foundation, Via della Signora 2, 20122 Milan, Italy

**Keywords:** motor vehicle crash, trauma, major trauma, high speed, energy trauma

## Abstract

In industrialized countries, high energy trauma represents the leading cause of death and disability among people under 35 years of age. The two leading causes of mortality are neurological injuries and bleeding. Clinical evaluation is often unreliable in determining if, when and where injuries should be treated. Traditionally, surgery was the mainstay for assessment of injuries but advances in imaging techniques, particularly in computed tomography (CT), have contributed in progressively changing the classic clinical paradigm for major traumas, better defining the indications for surgery. Actually, the vast majority of traumas are now treated nonoperatively with a significant reduction in morbidity and mortality compared to the past. In this sense, another crucial point is the advent of interventional radiology (IR) in the treatment of vascular injuries after blunt trauma. IR enables the most effective nonoperative treatment of all vascular injuries. Indications for IR depend on the CT evidence of vascular injuries and, therefore, a robust CT protocol and the radiologist’s expertise are crucial. Emergency and IR radiologists form an integral part of the trauma team and are crucial for tailored management of traumatic injuries.

## 1. Introduction

Major trauma is defined as an injury or a combination of injuries that are life-threatening and could be life changing because they may result in long-term disability [1].

Different conditions may cause major trauma, particularly high energy trauma, which is determined by deceleration, sudden impact or compression injuries [2,3] at speeds above 65 km/h in motor vehicle accidents (>45 km/h in motorcycle accidents) [4], following a fall from a height greater than 3 m or after sustaining crush injury between heavy objects [5].

Major trauma may produce unstable injuries, particularly vascular, which when becoming clinically apparent, may be so severe that treatment options are limited. This is the reason why an early and complete imaging approach is of paramount importance [6].

**Figure 1:**
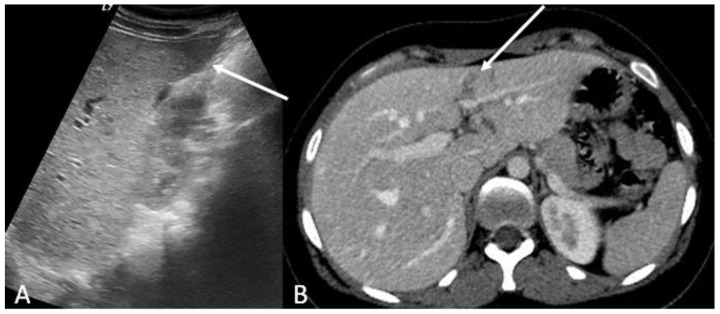
US (**A**) and enhanced-CT (**B**, venous phase) of a 32 year old male who sustained major trauma. US scans of the liver shows a subtle hypoechoic area (**A**, arrow). Enhanced-CT allows exhaustive evaluation of the suspected liver injury, depicting the whole extension of the liver laceration (**B**, arrow) and excluding the presence of vascular injuries, thus allowing safe conservative management of the patient.

In unstable patients undergoing major trauma, imaging approaches consists of FAST (Focused Assisted Sonography for Trauma) or, even better, the more comprehensive E(Extended)-FAST, as well as performing chest and pelvic X-rays. In stable or stabilized patients, whole-body CT (WBCT) has a pivotal role in the diagnosis of traumatic injuries showing high sensitivity and specificity in their detection and grading (Figure 1) as many guidelines, from North America and Europe, underline [7,8,9], it is proposed as first line comprehensive examination.

We hereby examine the crucial points regarding the indication, modality and role of CT in major trauma patients.

## 2. Indications

The main issue is to properly select patients that require CT evaluation after trauma to avoid imaging overutilization [10,11,12]. The optimal identification of the patient cohort for CT scanning remains a challenge, and up to 39–47% of patients undergoing a scan may have no injuries [13]. The choice is simpler when there is a combination of compromised vital parameters, severe trauma mechanisms and clinical examination findings in keeping with severe injuries [12]. More difficult, though, is the decision to perform a CT after high energy impact when physical examination is normal [14,15]. Debate continues about the risk-benefit ratio of systematic WBCT when no injuries are clinically suspected. In this cohort of patients, WBCT does not seem to change patient management and hence should not be performed routinely [16]. On the other hand, there is a progressive increase in technology utilisation due to its greater availability, and an increase in the number of emergency department admissions for trauma. This promotes the adoption of CT scans, considering the importance of the negative predictive value in shortening the patient’s hospital stay and increasing the physician’s level of certainty to manage and discharge patients [7,17,18,19,20].

## 3. CT Equipment

Nowadays, CT technology consists of a multislice-spiral CT between 4- and 320-slice CT [21]; tomography starting from 64-slices are preferred in trauma centres offering higher quality examinations [21]. Furthermore, in new technology development, efforts are made to reduce radiation exposure while maintaining good image quality i.e., through iterative reconstruction [22,23,24,25,26] or tube current modulation [23,26,27]. With iterative reconstruction techniques, radiation exposure can be reduced significantly [22,23,27], with an effective dose occasionally under 10 mSv for a WBCT scan [22,24,25]. Another option to reduce the radiation dose is the adoption of dual-energy CT, allowing the possibility of virtual noncontrast (VNC) images [28].

## 4. Timing of CT

The improvement in speed and accuracy of multidetector CT (MDCT) and increased availability of CT scanners in or near the trauma room have made immediate total-body CT feasible as a diagnostic tool in the initial assessment of trauma patients in several institutions, thus reducing time to reaching a diagnosis in life-threatening injuries [14,18,19,20,29]. Furthermore, in institutions where CT scanners have been introduced in trauma resuscitation rooms, a reduction in patient transportation time for CT examination was observed with ultimate reduction in time to control bleeding and a total decrease in mortality from exsanguination [12,30,31,32,33].

## 5. CT Protocol

The CT protocol to be adopted in polytrauma patients is still not standardized across institutions. Following the acquisition of an unenhanced scan of the head [15], a variety of protocols can be found in the available literature for body imaging, which differ in timing acquisition and the number of phases [34,35,36].

The monophasic protocol consists of a single CT acquisition after intravenous (IV) administration of contrast medium (CM) from neck to pelvis and preceded by an unenhanced scan of the head.

Multiphasic CT protocol includes a noncontrast scan of the head, followed by arterial and venous phases extending from the neck to the pelvis, with a single bolus and two separate acquisitions.

The split-bolus CT protocol consists of a single pass through the CT gantry after IV injection of two or three boluses (arterial and portal venous) of CM given sequentially, with a time delay or saline bolus in between. The sequential contrast boluses result in a single acquisition, reflecting the combination of arterial and portal venous phases (and potentially a urinary excretory phase).

Among the above, a multiphasic protocol should be considered the “optimal” CT protocol to be adopted initially and in follow up of high-energy trauma. The goal would be early detection and detailed characterization of injuries that may affect the patient’s treatment and prognosis, with a high degree of sensitivity and specificity, especially in vascular injuries which may require immediate intervention [34,37]. As drawbacks multiphasic protocols have a higher radiation dose compared with others and also a wide series of images that need to be interpreted in a short time, thus with a major risk of error [38].

Monophasic and split-bolus CT protocols may not allow adequate identification and characterization of vascular injuries such as pseudoaneurysms, arterial injuries and dissections, which may be masked by the timing of acquisition. Furthermore, the acquisition of only one post contrast phase does not allow accurate estimation of the volume of active bleeding present, neither does it precisely define the arterial or venous origin of injury [39].

The acquired volume of the CT examination in polytraumatized patients usually extends from head to pelvis. However, if vascular injury is suspected, such as in open or distal limb fractures, the entire upper or lower limb maybe included in the study [40,41] and it is usually easier to include both lower limbs in the CT examination [42]. On the contrary, when imaging upper limbs, one of the arms should be selected and positioned in full adduction to the trunk [42]. A multiphasic CT protocol is also suggested for limb examination to properly detect and characterize vascular injuries [42,43].

Even if it has been proven that the maintenance of a standard protocol for whole-body CT after polytrauma increases the probability of survival, there is the impression that the number of patients with minor injuries who undergo WBCT has increased [44]. In an attempt to limit the excessive dose exposure, the European Society of Emergency Radiology (ESER), made a recent proposal to consider at least two different WBCT protocols: the Time/Precision Protocol (multiphasic CT study) that should be preferred for polytrauma patients with life-threatening injuries or hemodynamically unstable conditions, and the Dose Protocol (split bolus) which is preferred for polytrauma patients who do not have obvious life-threatening injuries or are hemodynamically unstable [11,44].

## 6. Injury Classifications

To standardize the description and the communication of traumatic injuries, the American Association for the Surgery of Trauma (AAST) produced several lists of organ injury scaling that are constantly updated and online available [45].

## 7. Importance of Detection of Vascular Injuries

**Figure 2:**
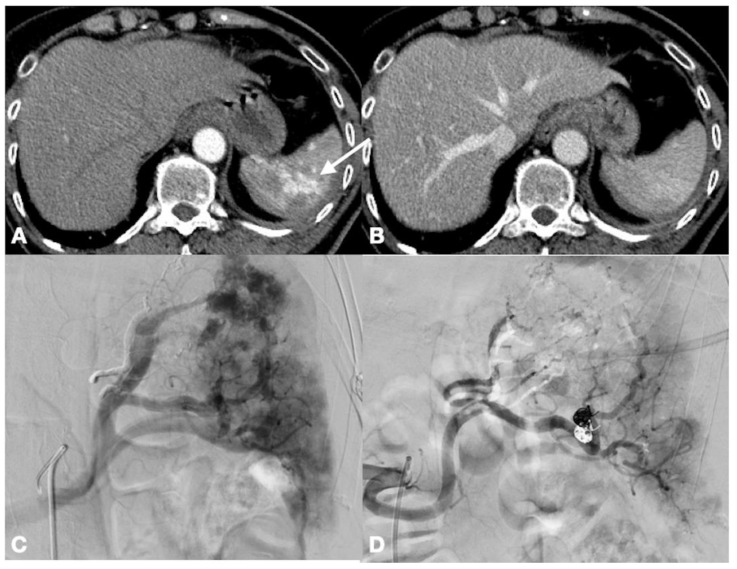
Enhanced-CT of a 56-year old male who sustained major trauma. Arterial (**A**) and portal venous phase (**B**). There is a small volume hemoperitoneum and multiple contained vascular injuries (**A**, arrow) that can be seen only in the arterial phase. The patient underwent angiography which confirmed CT findings (**C**), followed by successful embolization (**D**).

**Figure 3:**
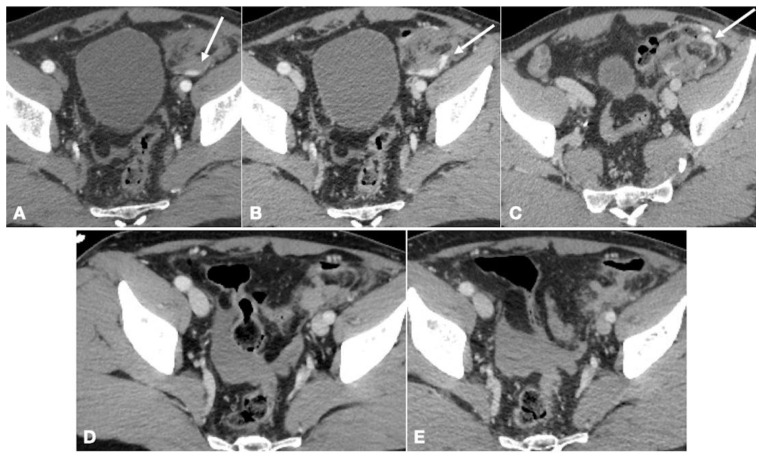
Enhanced-CT of a 54-year old male involved in major trauma (car accident). The multiphasic CT study allowed characterization of bleeding as arterial in origin, as seen on the arterial phase (**A**, arrow). Contrast extravasation persisted in the subsequent two phases (**B**,**C**, arrows). The patient then underwent angiography and embolization with absorbable material. Follow-up CT performed at 1st (**D**) and 4th (**E**) day after embolization showed no signs of bowel wall necrosis.

Acute vascular injuries are the second most common cause of fatality in patients with multiple traumatic injuries. Thus, prompt identification and management are essential for patient survival. CT has replaced catheter angiography as the primary screening study due to its high sensitivity in detecting], characterizing and grading vascular injuries and, therefore, only selected patients with specific indications for treatment are managed by IR [46,47] (Figure 2 and Figure 3).

**Figure 4:**
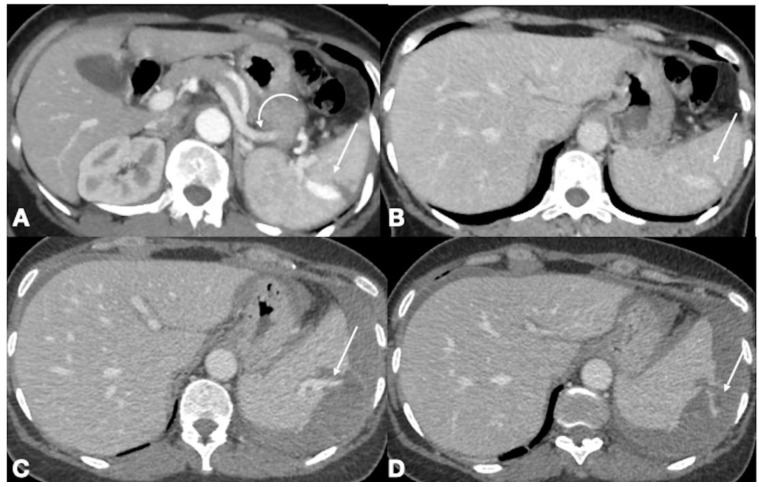
Enhanced-CT acquired in a 43-year old male who sustained major trauma (motor vehicle accident). Admission CT acquired in arterial (**A**) and portal venous (**B**) phases shows the presence of a contained vascular injury (pseudoaneurysm) within the splenic laceration (**A**, arrow). The pseudoaneurysm is associated with an arterio-venous fistula, demonstrated by early opacification of the splenic vein (curved arrow) in the arterial phase, synchronous with that of the splenic artery. The pseudoaneurysm is faintly seen in the following portal venous phase (**B**), and the arterio-venous fistula is not identifiable in this phase. The patient was scheduled for angiography and embolization but whilst awaiting the procedure, the vascular injury caused a spontaneous splenic rupture with active extrasplenic bleeding (**C**, arrow) which increased in the subsequent phase (**D**, arrow). Figure 4A was presented in the poster C-12530 Splenic Emergencies: value of US exploration for the diagnosis at ECR 2020.

Vascular injuries that can be identified range from minimal to major lesions: from arterial spasm and thrombosis, intimal tear, intramural hematoma, pseudoaneurysm (Figure 2), arteriovenous fistula (Figure 4) to active bleeding (Figure 3) [47]. Their prompt detection is crucial as nonbleeding injuries may also cause problems that become manifest hours, days or years after trauma. For example, arterial thrombosis may lead to organ ischemia, liver arterio-portal fistulas may lead to portal hypertension, and splenic arteriovenous fistulas may result in “spontaneous” splenic rupture (Figure 4).

The detection and characterization of active bleeding assume importance in terms of management, as not all active bleeding injuries require operative management in an emergency setting. Indeed, minor active bleeding, especially if intraparenchymal and of venous origin, may be self-limiting and managed conservatively [34] On the other hand, it is necessary in single or multiple arterial injuries to recognize and point out the urgency of the injury and feasibility of intervention in order to guarantee proper patient management.

In a recent study examining the effect of early door-to-CT time and door-to-control of bleeding time on mortality in patients with severe blunt trauma, the authors concluded that earlier time to hemostasis, including surgery and angioembolization, was independently associated with a decrease in mortality. This suggests that “time is blood” could be proposed as a standard for trauma management and designed to shorten time to control life threatening bleeding and reduce mortality in patients with severe trauma [48].

## 8. Thoraco-Abdominal Parenchymal Injuries

Pulmonary and intra-abdominal parenchymal injuries are exhaustively identified and graded using contrast enhanced CT [14]. A detailed grading system helps in patient risk stratification and proper management; preferably non-operative.

Indeed, surgical treatment as the commonest therapeutic strategy for solid organ injuries due to blunt trauma has evolved, and it is currently considered a better option to adopt conservative treatment aiming to preserve the injured organ as much as possible, with increasingly satisfactory results [49,50].

**Figure 5:**
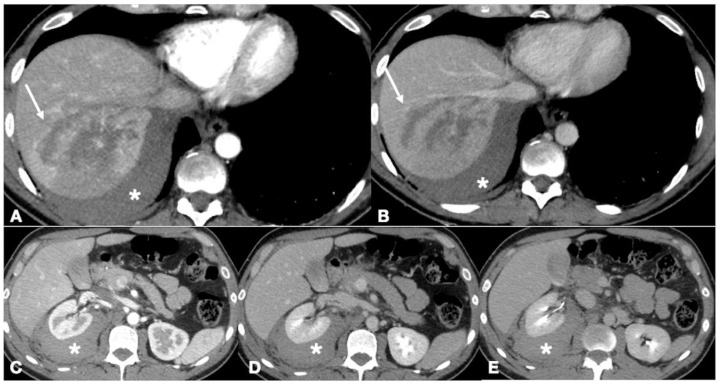
Enhanced-CT of a 37-year old male with multiple injuries due to high energy blunt trauma (car accident). The CT study demonstrates hemoperitoneum (**A**,**B**, asterisks), liver lacerations (**A**, arterial phase, arrow; **B**, portal venous phase, arrow) and a perirenal hematoma (**C**–**E** asterisks). The availability of multiple phases excluded the presence of active bleeding or active urine extravasation. The patient was managed conservatively.

Nonoperative treatment is now the first adopted strategy in hemodynamically stable patients with blunt trauma (Figure 5), and operative treatment is reserved to those with major kidney injury and urine leak, pancreatic injury encompassing the main pancreatic duct, bowel perforation, or in cases of conspicuous active venous bleeding [51,52,53,54,55,56,57,58].

Pancreatic and bowel injuries are particularly subtle and may become radiologically manifest several hours after trauma [56,59,60]. However, among the imaging methods, CT has the highest sensitivity for the detection of the traumatic injuries [59,61,62].

## 9. The Role of the Radiologist within the Trauma Team

In the emergency department (ED), complex trauma care requires strong inter-professional teamwork and resource management. Emergency radiologists have an active role in the emergency medical team interacting closely with emergency physicians and surgeons for management of critically ill patients [21].

The technological improvement of MDCT has led to a greater applicability of CT in trauma setting, reducing the time taken for CT scanning and promising high diagnostic accuracy even in subtle but significant injuries; thus, improving patient management.

In view of the increasing evidence of potential benefits in performing immediate total-body CT, several institutions have installed CT scanners in their trauma resuscitation rooms to eliminate transportation time and reduce diagnostic time to a minimum [33].

Furthermore, considering that rapid control of bleeding is pivotal in the management of the polytraumatized patient, and recent advances in IR have led to fast and minimal invasive treatment of vascular injuries, the most recent novel approach suggests a hybrid emergency room system in which prompt surgical management for both head and trunk injuries is also feasible [33].

## 10. Conclusions

In conclusion, the latest innovations in radiological systems have drastically changed the management of polytraumatized patients and led to prompt diagnosis, enabling speedy and timely treatment to reduce patient mortality [63].
